# Gender-based violence and its relationship to the mental health of female university students

**DOI:** 10.3389/fsoc.2025.1597261

**Published:** 2025-09-24

**Authors:** Kenia Laz-Figueroa, Brizeida Raquel Hernández Sánchez, José Carlos Sánchez García, Fabricio Guevara-Viejó, Valeria Molina-Molina

**Affiliations:** ^1^Universidad Técnica de Babahoyo, Babahoyo, Ecuador; ^2^National Research System (SNI), University of Salamanca, Salamanca, Spain; ^3^Centro de Estudios Estadísticos, Universidad Estatal de Milagro (UNEMI), Milagro, Ecuador; ^4^Facultad de Ciencias de la Salud, Universidad Espíritu Santo (UEES), Samborondón, Ecuador

**Keywords:** gender violence, mental health, university students, psychological abuse, risk factors

## Abstract

**Introduction:**

Gender-based violence (GBV) poses a significant threat to women’s mental health, especially in university settings where structural dynamics of subordination may persist. In Ecuador, more than 64% of women over the age of 15 have experienced some form of violence, underscoring the need for contextual studies.

**Methods:**

This study used a quantitative, cross-sectional design with a random sample of 380 female students from the Psychology program at the State University of Milagro (UNEMI). Two instruments were used: the Dating Abuse Questionnaire (DAQ) to assess five types of violence (psychological, physical, economic, sexual, and sociocultural) and the SCL-90-R inventory to measure mental health symptoms. Exploratory factor analyses and ANOVA tests were performed.

**Results:**

The results showed a high prevalence of psychological violence, with significant correlations between it and symptoms of paranoid ideation, anxiety, depression, and interpersonal sensitivity. The ANOVA analysis showed that students exposed to violence (psychological, physical, economic, sexual) had significantly higher levels of psychological symptoms than those who did not report experiences of violence. Sociocultural influence showed a weaker association with symptoms.

**Discussion:**

The findings confirm that GBV acts as a chronic stressor that negatively impacts the mental health of female university students. The need to implement institutional interventions and public policies that promote safe, inclusive, and violence-free academic environments is highlighted.

## 1 Introduction

Gender-based violence (GBV), especially against women, has been a topic of interest to researchers because of its profound psychological, physical and social implications for the victims. These violent acts not only endanger women’s lives, but also negatively affect their social and economic wellbeing. In Ecuador, gender violence is one of the most serious and structural problems in the country (ONU Mujeres, 2023).

The magnitude of this problem is reflected in alarming figures: 64.9% of women aged 15 years or older have experienced some type of violence, with a distribution of 35.4% in physical violence, 32.7% in sexual violence, 60% in psychological violence and 16.4% in economic or patrimonial violence (INEC, 2019). These statistics underscore a critical situation that demands immediate attention and action. Globally, violence against women between 15 and 49 years of age is also of concern, with rates ranging from 33 to 51% in regions such as Oceania, Asia and Africa, and 25% in Latin America and the Caribbean (La, 2021).

In the last 30 years, Ecuador has made significant progress in the fight against gender-based violence through the implementation of laws and public policies. This progress is due, in part, to the promotion of criminal justice as a fundamental mechanism for the protection of women’s human rights. Feminists have highlighted the importance of creating specific crimes related to violence against women (VAW) to facilitate accurate statistical tracking and ensure an adequate judicial response. Among the most notable initiatives, specialized VAW courts were established in 2013 to replace the police stations of the 1990s. In 2014, the new Penal Code criminalized several misdemeanors of VAW and femicides. Subsequently, in 2018, the “Comprehensive Law to Prevent and Eradicate Violence against Women” was enacted, a historic milestone in the country (Guamán Ortiz, 2023).

Despite these legislative advances, gender-based violence remains a persistent problem in Ecuador. Particularly in the university setting, women who face this type of violence can experience devastating effects on their mental health. Traumatic experiences can leave long-term emotional and psychological scars, affecting their quality of life and overall well-being. Universities play a crucial role in the formation and trans-mission of ideologies; however, they can also be spaces where norms that subordinate the feminine to the masculine persist. This type of structural violence perpetuates power asymmetries and contributes to violence against women.

The objective of this research is to evaluate the relationship between the types of violence received by female students of Psychology at the State University of Milagro (Unemi) and mental health symptoms. This approach seeks to make the situation visible in a specific context and to promote a culture of respect in the university environment.

The importance of addressing this problem lies in the alarming global and local statistics. Approximately 736 million women in the world, or one in three, have experienced physical or sexual violence by their partners. Moreover, these figures continue to rise, with particularly high incidence between the ages of 15 and 24 (La, 2021). In the Ecuadorian context, 64.9% of women over the age of 15 have experienced some type of violence (INEC, 2019). This reinforces the need to investigate and address the consequences of gender violence, especially in educational contexts such as universities.

The development of this research will contribute to the understanding of how different forms of violence impact the mental health of female university students and can serve as a basis for designing interventions and policies that promote a safer and more equitable environment.

## 2 Literature review

Forms of violence are not onetime incidents, but persist over time, even decades. Because of the sensitivity of the topic, violence is almost universally underreported. Gender-based violence is global in scope, occurring in both developed and developing countries regardless of dominant religion or political ideology. Among the forms it can take are intimate partner (or domestic) violence, rape (whether by acquaintances or family members or during wars and civil conflicts), trafficking for prostitution or other forced labor and debt bondage, physical and sexual injury for prostitution, sex-selective abortion and female infanticide or abandonment of girls, and female genital mutilation (Watts and Zimmerman, 2002). Studies have described that there is a growing body of research evidence suggesting that violence against women is highly prevalent, with an estimated one in three women worldwide experiencing some form of victimization in childhood, adolescence, or adulthood (García Navarro et al., 2020).

Violence against women (VAW) is commonly defined as any act of gender-based violence that results or is likely to result in physical, sexual, or psychological harm or suffering to women, including threats of such acts, coercion, or arbitrary deprivation of liberty, whether in public or private life (Falb et al., 2025). The term is rooted in sexism, patriarchy and gender inequality and, as such, is widely exercised against women in three inter-connected spheres: the family, the community and the state (Landeta-Bejarano et al., 2025). Research has shown that violence against women is a major cause of ill health among women and girls, the impact of which can be seen directly in death and disability due to injury, as well as indirectly through increased vulnerability to a range of physical and mental health problems (Devries et al., 2011).

In research by other contributors, the most common psychological symptoms due to gender-based violence are anxiety, depressive symptoms, low self-esteem, emotional lability, hypoactive sexual desire, ongoing fatigue, and insomnia. Sexual violence and victimization are associated with an increased risk of mental disorders (Watson and Bitsika, 2025). A threefold increase in the likelihood of depressive disorders, a fourfold increase in the likelihood of anxiety disorders, and a sevenfold increase in the likelihood of post-traumatic disorders have been reported for women who have experienced domestic violence (Agüero-Piazzini et al., 2024). In addition, systematic reviews of predominantly cross-sectional studies report consistent relationships between being a victim of domestic violence and battering and having mental disorders across the diagnostic spectrum for men and women, but because women are more likely to be victims, the population attributable fractions are higher for women (Schouler-Ocak and Brandl, 2022).

Globally, 35.6% of women have ever experienced either non-partner sexual violence or physical or sexual violence by an intimate partner, or both, with intimate partner violence (IPV) being the most common form (Monteiro et al., 2025). Worldwide, nearly one-third (30%) of all women who have been in a relationship have experienced physical and/or sexual violence by an intimate partner. However, variations between countries or regions are wide (9. 0.M.S OMDLS, 2021). Because of this, a recent review synthesizing data on intimate partner violence against women in Latin America focused exclusively on gender-based violence and highlighted the critical role of data from observatories in informing prevention and intervention strategies (Monteiro et al., 2025). All countries had a widespread prevalence of intimate partner violence (physical and sexual) among women who were ever married or in a relationship, and the results ranged from 17 to 53.3%. Only four of the countries included questions on non-partner violence in their population-based surveys. For example, in Haiti, 26.5% of women over 15 years of age had experienced physical violence by an aggressor during their lifetime. In the Dominican Republic, 20 and 10% of women over 15 years of age reported having suffered physical and sexual violence at some time in their lives, respectively; and in Peru, 39.5 and 19.7% of women reported having suffered physical violence at some time by their partner and by non-partners, respectively (Edeby and San Sebastian, 2021).

Gender-based violence (GBV) manifests in diverse interrelated forms and is recognized as a major public health and human rights issue, particularly in Latin America and the Caribbean. Recent studies emphasize not only the prevalence of GBV but also its multidimensional expressions, which include physical, sexual, psychological, economic, and sociocultural forms of violence (Tsapalas et al., 2021).

Recent scholarship has expanded the conceptual understanding of gender-based violence (GBV) by including the experiences of gender-diverse populations. Studies have emphasized the disproportionate vulnerability of LGBTQ+ and gender-diverse individuals to various forms of violence, including relationship abuse and cyber abuse, reflecting systemic social inequalities (Mickievicz et al., 2025; Gonzalez et al., 2025). These works also highlight the importance of considering intersectional factors, such as race, sexuality, and socioeconomic status, when analyzing GBV. Furthermore, theoretical advancements propose more inclusive frameworks for understanding gender, emphasizing the need to avoid binary reductions and recognize diverse gender identities within legal and social systems (Kirk-Giannini, 2025). This inclusive perspective enriches the understanding of GBV and promotes the development of more equitable prevention strategies.

This multidimensional framework of gender-based violence must be examined within the specific historical and cultural context of Ecuador. The colonial legacy has profoundly shaped contemporary social relations, reinforcing hierarchical structures based on race, gender, and socioeconomic status. These structural dynamics perpetuate systemic inequalities, particularly affecting women and marginalized populations, and contribute to the normalization of violence (Tsapalas et al., 2021; Segato, 2016).

Following Žižek (2008) theoretical perspective, a distinction is made between direct, interpersonal violence and systemic violence, which is embedded in cultural practices, institutional arrangements, and language. This analytical lens enables a broader understanding of how historical and structural factors sustain gender-based violence within Ecuadorian society.

- Physical violence: this type of violence is the most evident and encompasses any voluntary and aggressive act that causes or may cause harm to the woman’s body, whether or not it has visible results. This conduct involves hitting, slapping, pushing, pulling hair, kicking, burning, biting, strangulation, suffocation, stabbing, genital mutilation, use of weapons, restraints, induced abortions, torture, etc. The results can be fractures, wounds, bruises, contusions, hematomas and even death.
- Sexual violence: any attack on a woman’s sexual freedom by which she is forced against her will to endure acts of a sexual nature or to perform them, taking advantage of a situation of power, using deception, coercion, threats or the use of force. This attitude ranges from the use of jokes and sexual jokes, unpleasant comments, exhibitionism, unwanted sexual advances, unwanted touching to rape, incest, forced pregnancy, trafficking and exploitation in the sex industry.
- Psychological or emotional violence: action, usually of a verbal or economic nature, that causes or may cause psychological harm to women by acting on their decision-making capacity. It includes the use of control and communication mechanisms that undermine their psychological integrity, well-being, self-esteem or consideration, both public and private, in the eyes of others. It involves actions or omissions that comprise an extensive range of situations ranging from belittling, persistent verbal attacks, humiliation, shouting, threats, coercion, insults (Roca Monjo, 2011; INEC, 2014).

The experience of physical or psychological violence is associated with mental disorders. Among women who have suffered violence at the hands of their intimate partner, the prevalence of mental disorder is approximately 50% (Ludermir et al., 2008). Victims of violence are six times more likely than nonvictims to experience psychological problems (Romito et al., 2005).

Economic violence: refers to the inequality or state of control over access to shared resources. This implies deprivation of care and property of the cohabitant, control of economic resources, failure to comply with alimony payments in case of separation or divorce, denial of property rights, preventing access to a job, education or health care.

Structural/sociocultural violence: It is a fundamental concept for understanding inequalities and disparities in society. It is related to economic violence but includes intangible and invisible barriers that prevent women’s access to basic rights. It includes the denial of information inherent to fundamental rights and the power relations that keep them subordinate, in educational, decisionmaking or work centers. It refers to a nonexplicit form of violence, without blows, insults or threats (Roca Monjo, 2011), are forms of violence that are the product of norms, beliefs and cultural practices in a society. This form of violence is evidenced through discriminatory attitudes, harmful stereotypes, rigid gender roles, ethnic or racial prejudice, among others.

In the questionnaires applied in the present investigation, there are contributions from different collaborators, such as that of Sanchez and Ledesma (2009), whose objective was to apply the SCL90-R questionnaire in a clinical population of 570 people to provide local data for its respective use in Argentina and obtained a Cronbach’s alpha between 0.72 and 0.86. This study suggests that an exploratory factor analysis should be performed at this time to explore the underlying factors of the questionnaire.

On the other hand, the study by Gempp Fuentealba and Avendaño Bravo (2008) presented an adaptation of the Derogatis Symptoms Inventory in Chilean university students applied to a sample of 718 students (men and women), the results of the study indicated that the questionnaire has an acceptable performance, but it is necessary to explore the questionnaire further, as there were no differences between men and women. The study by Londoño et al. (2018) established the psychometric properties of the Derogatis 90 for the Colombian population applied to a sample of 214 people who went to consultation (men and women), the results concluded the structure with 36 items and seven factors of the nine factors of its original version: depression, obsession compulsions, somatization, anxiety, hostility, phobic anxiety and paranoid ideation.

Finally, the study by Cebreros (2022) used a sample of 1,455 participants (men/women), and concluded that the structure of the questionnaire does not coincide with the original, in fact, there were difficulties in validating the original model, preventing a clarification of its dimensions. This author suggests that it is more convenient to use the questionnaire to measure general psychological distress.

Vela et al. (2020) evaluated the psychological symptoms of the SCL-90-R. addressed to both sexes, showing a high internal consistency which confirms its reliability, it was applied for a sample of 767 people (women 574–187 men) during the confinement by COVID 19 in Mendoza-Argentina.

Furthermore, it kept intact the predominant factors. Colque Casas (2020) presented as an objective to determine the psychological consequences of women victims of violence, for a sample of 108 women who made a complaint against their partner in Callao, the results of the study concluded that the symptoms present in the victims of abuse are: depression, obsession—compulsion, anxiety and somatization.

Finally, Burbano-Larrea et al. (2023) determined the prevalence of violence in intimate partner relationships in students of the careers of Psychopedagogy, Educational Psychology of the University to a sample of 263 students of the aforementioned. This study provides relevant information to understand and address violence in affective relationships among young university students. The results conclude that the most prevalent forms of violence are psychological violence and sexual violence, and the cultural factor is attributed as the main contributor to the continued subordination and domination of one gender over the other.

According to the study by Lazarevich et al. (2013) which aimed to evaluate dating violence, symptomatology and self-esteem and to study the relationship between the variables. This study was addressed to a sample of 729 students. Its results showed that there is a high prevalence of violence in young university students, especially verbal-emotional violence (75%). It was found that violence is bidirectional, both men and women exercised and suffered violence.

Osorio-Guzmán (2014) in his study performs an analysis of the psychometric properties of the Maltrato en el Noviazgo Questionnaire in a sample of Mexican female students of middle and high school level. According to their results they concluded that the instrument has an internal consistency of *α* = 0.95; through a confirmatory factor analysis establishing 5 factors to reliably assess dating abuse.

Based on the study by Guzmán et al. (2021) with the purpose of analyzing the types of dating abuse, applied to a sample of 715 high school students in Mexico. The study concludes that the prevalence is given in psychological and social mistreatment.

In Ecuadorian regulations on VAW are largely the result of a global and regional shift towards criminal justice to address gender inequalities, often framed as obstacles to development and security. This shift is most evident beginning in the 1990s in relation to the mainstreaming of international discourses that frame VAW as a human rights and development issue (Bedford, 2020).

The first National Survey of Family Relations and Gender Violence against Women in Ecuador was conducted by the National Institute of Statistics and Census (INEC) in 2011 and included 18,800 women aged 15 years and older. The results showed that 60% of women had experienced some form of VAW (including psychological, physical, sexual and economic violence), with psychological violence (53.9%) being the most common type of violence. Among women who had experienced physical violence, it was most often carried out by an intimate partner (INEC, 2014). In INEC (2019) conducted a second survey, not only to respond to the obligation to collect information on gender-based violence assigned by the Institute in the 2018 Law, but also to provide up-dated evidence for the indicators of the National Development Plan and the 2030 Agenda for Sustainable Development related to gender-based violence.

## 3 Materials and methods

The approach of this study was quantitative, with a probability sample and a cross-sectional design given that the data were collected at a specific time interval.

### 3.1 Participants

The population of interest in this study consisted of female students in the Psychology program at Milagro State University (3,028 students). The sample was determined by using the finite population equation, obtaining a sample of 380 female students.

### 3.2 Sample size calculation

The sample size was determined using the formula for finite populations, with the aim of obtaining a statistically representative sample:


$$n=\frac{N\cdot Z^{2}\cdot p\cdot (1-p)}{e^{2}(N-1)+z^{2}\cdot p\cdot (1-p)}$$

Where:

- *N* = 3,028 represents the total number of enrolled students,
- *Z* = 1.96 corresponds to the 95% confidence level,
- *p* = 0.5 was assumed to maximize the required sample size,
- *e* = 0.05 represents the accepted margin of error.

### 3.3 Sampling procedure

After calculating the required sample size, a simple random probability sampling method was applied to select participants. This method ensured that every active student had an equal probability of being selected. Access to the student database was granted by the Academic Secretariat of UNEMI, maintaining confidentiality at all times. Eligible participants received a formal invitation via their institutional email, which included a detailed information sheet explaining the study objectives, voluntary participation, implied consent, and a link to the anonymous online questionnaire.

In total, 600 invitations were distributed to eligible students, of which 380 fully completed responses were received, resulting in an overall response rate of 63.3%. Only completed questionnaires meeting the established quality criteria were included in the final analysis.

### 3.4 Instruments

For the study, a digital survey was conducted via email among students in the Psychology program at the State University of Milagro (UNEMI). Two instruments were used to collect data.

The first instrument was the SCL-90-R Symptom Inventory modified by Derogatis (Casullo and Pérez, 2008), which consists of 90 items scored on a 5-point Likert scale (0 = not at all, 4 = extremely). This instrument was used to measure the mental health variable (symptomatology), assessing psychological symptoms in multiple dimensions, such as depression, anxiety, somatization, and others.

The second instrument was the Dating Abuse Questionnaire (DAQ), which consists of 61 items that assess five different dimensions of dating violence: psychological, physical, economic, sexual, and sociocultural abuse. This questionnaire allowed us to identify both the presence and level of abuse experienced by the participants.

The estimated time needed to complete the entire set of questionnaires was approximately 20–25 min, based on the results obtained in a preliminary pilot test with 15 psychology students. The survey was designed to be easy to use, minimizing cognitive load, and included automatic prompts to ensure that it was completed before submission.

#### 3.4.1 Data collection instruments

It presents five factors or dimensions that are distributed into: physical, psychological, sexual, economic and sociocultural influence (Table 1).

- *Psychological mistreatment:* Psychological damage is evaluated, involving actions to dominate or socially isolate the partner from family, friends, etc. It also involves exercising control by imposing control over how to dress, comb one’s hair or behave in public through verbal aggression or simply silence (Guzmán et al., 2012).
- *Physical mistreatment:* This factor evaluates the physical action or harm that the aggressor exerts against the partner involving: punching, pinching, attempted strangulation, among other actions (Paravic-Klijn et al., 2018).
- *Economic mistreatment:* this dimension is aimed at analyzing acts that have the purpose of forcing or economically depending on the partner of her aggressor. So that her partner does not let her work, controlling the victim’s economic resources (Guzmán et al., 2012).
- *Mistreatment: Sexual:* The objective is to analyze actions of harassment, sexual exploration, sexual violation that are carried out without the consent of the partner, violating her rights in a way that affects the victim’s integrity, self-esteem and generates frustration and self-doubt (Sánchez Ramón et al., 2004).
- *Social Influence:* Analyze those preconceived and discriminatory social roles directed at the female gender (Guzmán et al., 2012) (Table 2).

**Table 1:** Dating abuse questionnaire dimensions.

Dimensions	# Items	Items
Psychological abuse	28 ítems	1, 2, 3, 4, 5, 6, 8, 10, 12, 13, 14, 17, 18, 19, 23, 24, 25, 26, 29, 30, 32, 33, 36, 39, 40, 43, 44, 51.
Physical abuse	9 ítems	21, 28, 34, 38, 41, 45, 50, 52, 53.
Economic abuse	7 ítems	7, 16, 35, 46, 47, 48, 49.
Sexual abuse	9 ítems	9, 11, 15, 20, 22, 27, 31, 37, 42.
Socio-cultural influence	8 ítems	54, 55, 56, 57, 58, 59, 60, 61.
Psychological abuse	28 ítems	1, 2, 3, 4, 5, 6, 8, 10, 12, 13, 14, 17, 18, 19, 23, 24, 25, 26, 29, 30, 32, 33, 36, 39, 40, 43, 44, 51.

*Scaling:* for this questionnaire, a scaling table was established to measure the presence or absence of abuse in the Dating Abuse questionnaire; a discretization was made for each of the types of abuse by the “grouping by container” method, as shown in Table 2.

**Table 2:** Measurement scaling.

Type of abuse	Ranges	Description
Psychological mistreatment (M_Psic)	26–62	Absence of abuse
Physical abuse (M_Physical)	63–99	Presence of abuse
	8–23	Absence of maltreatment
	24–39	Presence of abuse
Economic abuse (M_Econ)	6–17	Absence of abuse
	18–29	Presence of abuse
Sexual abuse (M_Sexual)	13	Absence of abuse
	22	Presence of abuse
Social influence	5–9	Absence of maltreatment
	10–14	Presence of abuse

#### 3.4.2 Data collection procedure

It evaluates patterns of symptoms present in individuals. It consists of a total of 90 items (Table 3) with their respective Likert scale to evaluate 5 points from 0 to 4: where nothing (0), very little (1), a little (2), quite a lot (3), a lot (4). The measures of this questionnaire and other items for the present study are:

**Table 3:** Underlying dimensions of the SCL-90 R symptom inventory.

Dimensions	# Items	Items
Somatizations	(12 ítems)	1, 4, 12, 27, 40, 42, 48, 49, 52, 53, 56, 58.
Obsessions and compulsions	(10 ítems)	3, 9, 10, 28, 38, 45, 46, 51, 55, 65.
Interpersonal sensitivity	(9 ítems)	6, 21, 34, 36, 37, 41, 61, 69, 73.
Depression	(13 ítems)	5, 14, 15, 20, 22, 26, 29, 30, 31, 32, 54, 71, 79.
Anxiety	(10 ítems)	2, 17, 23, 33, 39, 57, 72, 78, 80, 86.
Hostility	(6 ítems)	11, 24, 63, 67, 74, 81.
Phobic anxiety	(7 ítems)	13, 25, 47, 50, 70, 75, 82.
Paranoid ideation	(6 ítems)	8, 18, 43, 68, 76, 83.
Psychoticism	(10 ítems)	7, 16, 35, 62, 77, 84, 85, 87, 88, 90.
Additional items	(7 ítems)	19, 44, 59, 60, 64, 66, 89.

##### 3.4.2.1 Global severity index (GSI)

This is an indicator of the current level of severity of distress. It is obtained with the total sum divided by the number of responses. Subsequently, a “grouping by containers” is performed to classify them into 3 levels (normal, moderate and severe).

##### 3.4.2.2 Total positive symptoms (PST)

Sum of items that scored 0 or not at all. Total sum divided by the positive symptoms.

##### 3.4.2.3 Positive symptomatic distress index (PSDI)

Multiplication of the GSI by the number of items, divided by the total number of positive symptoms (PST).

### 3.5 Data quality assurance

To ensure the quality and reliability of the data collected via online surveys, several measures were implemented. Participants were first presented with a clear informed consent statement before accessing the survey, in accordance with the ethical principles outlined in the Declaration of Helsinki (World Medical Association, 2013). Responses completed in under 10 min were excluded to minimize inattentive or random responses. Built-in validation mechanisms prevented the submission of incomplete surveys. A preliminary pilot test was conducted to ensure the clarity and comprehensibility of the survey items. Additionally, a single reminder email was sent to avoid response fatigue or duplicate submissions. These quality control measures were designed to reduce response bias and enhance data validity.

### 3.6 Analysis

The Spss software was used to analyze the results of the study of gender violence and its relationship with the mental health of female university students. Among the analyses performed were the KMO index (Kaiser-Meyer-Olkim), exploratory factor analysis of the abuse questionnaire and a series of one-factor ANOVAs to visualize the difference between groups with presence and absence of abuse.

## 4 Results

The results obtained are distributed in different sections, addressing both construct adequacy and reliability analyses, exploratory factor analysis (EFA), ANOVA analysis of variance and correlational analyses.

### 4.1 Abuse questionnaire

First of all, before carrying out an EFA, it is necessary to evaluate the KMO index (Kaiser-Meyer-Olkim), which analyzes the suitability of the data for carrying out the exploratory factor analysis. It is evident that the KMO value obtained in the study is 0.894. As indicated by Shrestha (2021), those values between 0.8 and 1.0 are considered suitable for performing EFA. Subsequently, Bartlett sphericity test, which allows us to evaluate the relationship between variables, yielded a result of *p* = 0.000.

Once the corresponding Bartlett and KMO tests were performed, we proceeded to perform the exploratory factor analysis. The AFE has the purpose of exploring the corresponding factors of the questionnaire where 5 factors corresponding to the types of mistreatments of violence were established, applying a method of extraction of principal components and promax. The principal components method aims to identify the number and composition necessary to summarize in a set of observed variables [44]. In addition, Martínez and Sepúlveda mention that the promax rotation is applied with the purpose of making the factor loadings as close as possible to the ideal structure (p. 205).

Table 4 shows the results of the exploratory factor analysis (EFA), which indicate the factor loadings of each item on its respective latent factors. Factor loadings represent the correlation between each observed item and the underlying construct that is intended to be measured. Higher factor loadings indicate a stronger relationship, suggesting that the item contributes more effectively to explaining the corresponding factor. For example, item M1 showed a factor loading of 0.710 on the “Psychological Abuse” factor, indicating a substantial association with this construct. According to psychometric standards, factor loadings are normally considered acceptable when they exceed 0.30, moderate when they exceed 0.50, and strong when they exceed 0.70 (Hair et al., 2019). In this analysis, most of the retained items showed loadings within a satisfactory range (0.434–0.865), supporting the factorial validity of the instrument. Items below the 0.30 threshold were excluded from the final model to maintain structural consistency and interpretive clarity.

**Table 4:** Exploratory factor analysis.

Structure matrix					
Item	M_Psic	M_Físico	M_Econ	M_Sexual	Inf_Social
M 1	0.710				
M 2	0.621				
M 3	0.594				
M 4	0.719				
M 5	0.628				
M 6	0.496				
M 8.	0.532				
M 10	0.825				
M 12	0.839				
M 13	0.593				
M 14	0.692				
M 17	0.822				
M 18	0.865				
M 19	0.742				
M 24	0.529				
M 25	0.801				
M 26	0.806				
M 29	0.588				
M 30	0.359				
M 32	0.680				
M 33	0.705				
M 36	0.751				
M 39	0.483				
M 40	0.434				
M 43	0.732				
M 51	0.752				
M 21		0.814			
M 28		0.881			
M 34		0.863			
M 38		0.837			
M 41		0.804			
M 45		0.644			
M 50		0.773			
M 53		0.762			
M 7.			0.461		
M 16			0.474		
M 35			0.580		
M 47			0.477		
M 48			0.610		
M 49			0.597		
M 9				0.485	
M 11				0.300	
M 20				0.334	
M 22				0.313	
M 37				0.444	
M 56					0.595
M 57					0.583
M 59					0.511
M 60					0.572
M 61					0.672

^1^Extraction method: principal component analysis. Rotation method: Promax (Hair et al., 2019).

M_Psic (psychological Abuse), M_Physical (physical abuse), M_Econ (economic abuse), M_Sexual (sexual abuse), Inf_Social (social influence).

In the process of the exploratory factor analysis of the dating abuse questionnaire, several items were eliminated because they did not meet the loadings greater than 0.30 in the case of questions 15, 23, 27, 31, 42, 44, 46, 46, 52, 54, 55, 58.

Subsequently, from the AFE analysis, the reliability of the maltreatment questionnaire was checked, showing Cronbach’s Alpha score of 0.968. An internal consistency >0.9 which, means that it has an excellent internal consistency (Barrera Ortiz et al., 2015; Caycho-Rodríguez, 2017). In case *ɑ* < 0.5 is unacceptable; <0.5 is poor, ≥0.6 is questionable, ≥0.7 to >0.8 is acceptable, *ɑ* ≥ 0.8 to >0.9 is good and *ɑ* ≥ 0.9 is excellent (Vega Martínez et al., 2019; Sisniegas-Vergara et al., 2023).

### 4.2 Cuestionario de SCL—90 R

It is evident that the KMO value obtained in the study is 0.963 indicating a better adequacy than the maltreatment questionnaire and revealing that the AFE is pertinent to the data recovered. Similarly, Bartlett’s coefficient of sphericity rejects the hypothesis of equality between matrices and infers that there is sufficient correlation between the variables to continue with the analysis.

In the process of the exploratory factor analysis of the SCL-90-R questionnaire, several items were eliminated because they did not meet the loadings greater than 0.30 in the case of questions: 7, 8, 16, 35, 64, 75, 82, 85 and 2 dimensions (Psychoticism/Phobic anxiety) were eliminated, as shown in Table 5.

**Table 5:** Dimensions according to AFE.

Dimensions	# Items	Items
Somatizations	(11 ítems)	4, 12, 27, 40, 42, 48, 49, 52, 53, 56, 58.
Obsessions and compulsions	(11 ítems)	3, 9, 10, 28, 38, 45, 46, 51, 55, 65, 84.
Interpersonal sensitivity	(9 ítems)	6, 21, 34, 36, 37, 41, 61, 69, 73.
Depression	(15 ítems)	1, 5, 14, 15, 20, 22, 26, 29, 30, 31, 32, 54, 71, 79, 88
Anxiety	(16 ítems)	2, 13, 17, 23, 25, 33, 39, 47, 50, 57, 70, 72, 78, 80, 86, 87.
Hostility	(6 ítems)	11, 24, 63, 67, 74, 81.
Paranoid ideation	(6 ítems)	19, 44, 59, 60, 66, 89.
Additional items	(7 ítems)	19, 44, 59, 60, 64, 66, 89.

The reliability of the SCL-90-R questionnaire was tested, showing a Cronbach’s Alpha score of 0.988. An internal consistency >0.9 which, means that it has excellent values (Barrera Ortiz et al., 2015; Caycho-Rodríguez, 2017).

During the Exploratory Factor Analysis (EFA) exploration of both questionnaires, it was observed that certain questions might not be measuring a specific construct directly, and for this reason they were eliminated. Despite this initial consideration, the questions demonstrated excellent internal consistency, suggesting that, although the factorial grouping does not clearly reflect a single underlying construct for the questionnaires, the responses to the questions are highly consistent with each other.

### 4.3 Sociodemographic characteristics

On the other hand, the sociodemographic distribution informs us about the reality in which the study sample is found, with young women between 20 and 32 years of age, most of whom are single or married and live with their partner. Likewise, the results show that 60% of the women perceive that they have experienced violence at least once by their partner.

The high frequency of mistreatment among female students is reflected in Table 6, which reports the mean scores for each type of abuse. The Psychological Abuse dimension recorded a mean score of 39.63 with a standard deviation (SD) of 17.75, indicating it is the most prevalent form of abuse among the evaluated sample. The Physical Abuse dimension presented a mean of 9.83 (SD = 4.11), while Economic and Sexual Abuse dimensions recorded means of 7.84 (SD = 3.44) and 6.69 (SD = 2.72), respectively. Finally, the Sociocultural Influence dimension exhibited the lowest mean score of 6.10 (SD = 1.72), suggesting relatively lower frequency in the participants’ experiences.

**Table 6:** Descriptive statistics of the types of violence according to the Dating Abuse Questionnaire scale.

Dimensions	Mean	*σ*	*σ* ^2^	Skewness	Kurtosis
Psychological	39.629	17.750	315.062	1.476	1.094
Physical	9.839	4.108	16.874	3.959	20.245
Economic	7.847	3.449	11.892	2.411	6.114
Sexual	6.692	2.776	7.707	2.450	6.967
Sociocultural infl.	6.105	1.713	2.933	1.932	3.542

In order to evaluate the relationship between groups of people presenting symptoms of poor mental health and the presence or absence of abuse, an ANOVA test was performed, which shows that there are significant differences in the level of symptomatology between groups of people exposed to different types of abuse (Psychological, Physical, Economic and Sexual) compared to those who are not. For example, those individuals with the presence of psychological maltreatment have a significantly higher mean on the symptomatology scale (188.47) compared to those without this exposure (79.90). This supports the relationship between mental health problems and the presence of some type of maltreatment, in addition, the findings also strengthen that negative social influence does not influence symptomatology, since no significant differences were found in relation to this characteristic.

Finally, the results of Table 7 demonstrate statistically significant positive associations between different forms of maltreatment and mental health symptoms, assessed through Spearman’s correlation coefficients. The strongest correlation was observed between psychological maltreatment and paranoid ideation, with Spearman’s rho = 0.528, *p* < 0.001, indicating a moderate-to-strong relationship. Additionally, psychological maltreatment showed significant correlations with somatization (rho = 0.455, *p* < 0.001), interpersonal sensitivity (rho = 0.522, *p* < 0.001), and other symptom dimensions. In contrast, sociocultural influence presented weaker but still significant correlations, with the highest value observed for hostility (rho = 0.266, *p* < 0.001) and paranoid ideation (rho = 0.304, *p* < 0.001). These results confirm that direct forms of abuse, particularly psychological abuse, have a stronger association with mental health symptomatology.

**Table 7:** Spearman’s correlation coefficient.

Dimensions	SO	OB. C	SI	DE	AN	HO	I. P
Psych.	0.455**	0.475**	0.522**	0.503**	0.440**	0.500**	0.528**
Phys.	0.385**	0.363**	0.444**	0.421**	0.360**	0.362**	0.436**
Econ.	0.418**	0.448**	0.505**	0.484**	0.410**	0.446**	0.485**
Sex.	0.424**	0.424**	0.485**	0.463**	0.406**	0.456**	0.474**
Socioc. Infl.	0.278**	0.326**	0.224**	0.283**	0.259**	0.266**	0.304**

^1^Symptoms: somatization (SO), obsessions compulsions (OB. C), interpersonal sensitivity (S. I), depression (De), anxiety (AN), hostility (HO), paranoid ideation (I. P) (Hair et al., 2019).

**p < 0.001: Spearman correlation coefficients (bilateral).

## 5 Discussion

Psychological violence is defined as: behaviors, attitudes and communication styles based on humiliation, discrediting, control, hostile withdrawal, as well as domination and intimidation, denigration and jealous behaviors, which it describes as a domination behavior that is expressed through a dominant aggressive style (Novoa et al., 2016). Mondragón Barrera (2014) reported in his study for psychological maltreatment a strong correlation with symptoms of paranoid idea (0.528), symptoms of interpersonal sensitivity (0.522), depression (0.503), hostility (0.500), indicating that there is a considerable positive correlation. Regarding obsessive-compulsive (0.475) and somatization (0.455) represent an average positive correlation. Roca Monjo (2011) determined that women with psychological abuse evidenced symptoms of depression, obsessive-compulsion, anxiety and somatization. Exposure to violence can generate symptoms such as: anxiety and depression among others, and both variables are associated (Plazaola Castaño and Ruiz Pérez, 2004; Raya Ortega et al., 2004; Park Ryeong, 2017; Chandan et al., 2019). García Navarro et al. (2020) concluded that problems in cognitive processes have an impact on the quality of life (home, social and work) of women who are exposed to violence.

Apaza Zúñiga et al. (2022) showed that physical abuse has a moderate positive correlation with interpersonal sensitivity 0.440, paranoid ideation 0.436, and depression 0.421. Additionally, Campbell and Lewandowski (1997) identified other symptoms related to physical violence, such as stress, including irritable bowel syndrome. Alquaiz et al. (2023) highlighted that physical violence significantly affects selfesteem, which in turn impacts interpersonal sensitivity. This sensitivity manifests as feelings of personal inferiority in the victim. Their results indicated that higher self-esteem was associated with lower levels of physical abuse. Furthermore, Marsh et al. (2022) noted that individuals exposed to physical violence might develop psychotic experiences, emphasizing the need for public health policies to address this issue.

Economic abuse occurs when the perpetrator restricts or isolates the victim from the resources necessary for personal development, such as assets or money. Correlations ranging from 0.11 to 0.50 are considered moderate positive, while 0.51–0.75 indicate a strong positive correlation (Casullo and Pérez, 2008; Hair et al., 2019). According to the results, economic abuse showed a strong positive correlation with interpersonal sensitivity symptoms (0.505), while paranoid ideation symptoms 0.485 and depression 0.484 had moderate positive correlations. The effects of economic violence arise when the perpetrator fosters economic dependency in the victim, generating feelings of helplessness (Zabala Abarca, 2014). Authors have noted that economic violence is associated with psychological symptoms such as depression, anxiety, and suicidal ideation. Its presence increases the likelihood of these symptoms manifesting (Zabala Abarca, 2014; Antai et al., 2014; Kanougiya et al., 2021; Johnson et al., 2022) emphasized the role of economic violence in the psychological wellbeing of intimate partner violence survivors, demonstrating its significant impact on health. González Otero (2016) argued that individuals subjected to economic abuse lack personal autonomy, with abusers fostering devaluation through psychological abuse, which affects interpersonal sensitivity and, consequently, the victim’s self-esteem.

Alkan and Hüseyin (2021) described sexual abuse as coercing another person into sexual relations without prior authorization or consent. The results showed that sexual abuse has a moderate positive correlation with symptoms such as interpersonal sensitivity (0.485), paranoid ideation (0.485), and depression (0.484). Correlations between 0.11 and 0.50 are considered moderate positive (Casullo and Pérez, 2008; Hair et al., 2019). Individuals who have experienced sexual violence are associated with psychotic symptoms and others such as anxiety, depression, hostility, fear, posttraumatic stress disorder (PTSD), phobia, and psychosocial maladjustment (Alkan and Hüseyin, 2021; Agardh et al., 2012; Presaghi et al., 2015). The impact of sexual violence creates feelings of helplessness and low selfesteem in the victim, generating a sense of vulnerability to similar situations (Molina Rico et al., 2022).

The study results showed that sociocultural influence had a weak correlation with obsessive-compulsive symptoms (0.326) and paranoid ideation (0.304). This indicates a moderate positive correlation, ranging from 0.11 to 0.50. Similar findings were reported by Maguele et al. (2020), who indicated that sociocultural factors significantly influence intimate partner violence.

## 6 Conclusion

ANOVA and Spearman correlation analyses show a statistically significant relationship between exposure to various types of abuse (psychological, physical, economic, and sexual) and an increase in mental health symptoms. Specifically, individuals reporting experiences of abuse exhibit higher levels of symptomatology, including somatization, obsessions and compulsions, depression, anxiety, hostility, and paranoid ideation. This underscores the critical importance of considering abuse as a key determinant of mental health.

The significant relationship between various forms of maltreatment and a wide range of mental health symptoms suggests that maltreatment acts as a chronic stressor, triggering or exacerbating preexisting mental health disorders or inducing new psychological disorders. This relationship, supported by the existing literature, infers that stressful life events, especially those that are chronic and beyond the control of the individual, such as maltreatment in its various forms, have a profound impact on mental health.

On the other hand, unlike direct types of abuse, negative social influence does not have a strong impact on mental health symptomatology. While the correlations and ANOVA analyses indicate a statistically significant relationship, its magnitude is relatively smaller compared to that of abuse. This suggests that while social influence plays a role in mental health, the direct effects of abuse are more decisive in determining the levels of symptomatology observed in victims.

## Data Availability

The raw data supporting the conclusions of this article will be made available by the authors, without undue reservation.
